# Carbonation-Cured Cementitious Materials Incorporating Waste Rubber/Slag with Balanced Mechanical Strength and Microwave Absorption Performance

**DOI:** 10.3390/polym18161942

**Published:** 2026-08-07

**Authors:** Xuemin Zeng, Hao Zhang, Hongping Zhang, Pan He, Laibao Liu, Xian Jian, Xiaoshuang Shi, Youhong Tang, Qingyuan Wang

**Affiliations:** 1 State Key Laboratory of Metal Mining Safety and Disaster Prevention and Control, Maanshan 243000, China; zengxuemin935@163.com; 2 School of Metallurgical Engineering, Anhui University of Technology, Maanshan 243032, China; fengxu19821018@163.com; 3 School of Mechanical Engineering, Institute for Advanced Study, Chengdu University, Chengdu 610106, China; wangqy@scu.edu.cn; 4 Sichuan Provincial Engineering Research Center of Functional Development and Application of High Performance Special Textile Materials, Chengdu Textile College, Chengdu 611731, China; hepan-2009@163.com; 5 School of Materials and Chemistry, Southwest University of Science and Technology, Mianyang 621010, China; liulaibao@swust.edu.cn; 6 School of Materials and Energy, University of Electronic Science and Technology of China, Chengdu 610054, China; jianxian@uestc.edu.cn; 7 Failure Mechanics & Engineering Disaster Prevention and Mitigation, Key Laboratory of Sichuan Province, College of Architecture & Environment, Sichuan University, Chengdu 610065, China; 8 Institute for NanoScale Science and Technology, College of Science and Engineering, Flinders University, Adelaide, SA 5042, Australia

**Keywords:** waste rubber, cement-based microwave-absorbing materials, structural design, solid waste recycling, CO_2_ curing

## Abstract

Electromagnetic wave absorption ability and mechanical strength are critical performance metrics for cement-based microwave-absorbing materials. Enhancing electromagnetic wave absorption efficiency typically involves the incorporation of functional phases and optimization of pore structures. However, these modifications often introduce challenges, such as interfacial incompatibility between the functional phase and cement matrix, and reduced material density, which can compromise mechanical integrity. This study presents a structurally engineered, high-performance cement-based microwave-absorbing material fabricated from solid waste materials. By leveraging the poor interfacial compatibility between rubber powder and cement paste, the material achieves increased porosity, thereby improving impedance matching. Additionally, the presence of abundant dielectric and magnetic components in slag significantly enhances electromagnetic wave dissipation. Through the synergistic tuning of impedance matching and dissipation capacity, the cement-based microwave-absorbing material demonstrates a substantial improvement in electromagnetic wave absorption, with the absolute value of its reflection loss increasing by 2.8 times after CO_2_ curing. Furthermore, the application of CO_2_ curing technology facilitates the transformation of alkaline compounds such as Ca(OH)_2_ into CaCO_3_, resulting in notable gains in mechanical performance—compressive strength and flexural strength are elevated by 38% and 23%, respectively. This work not only achieves a balanced optimization of electromagnetic wave absorption and mechanical robustness in cement-based materials but also offers a sustainable pathway for the high-value utilization of industrial solid waste.

## 1. Introduction

With the rapid development of electromagnetic wave technologies and the widespread use of electronic devices, daily life is increasingly exposed to potential electromagnetic risks. These risks include interference with precision instruments, leakage of sensitive information from critical facilities such as data centers, and possible adverse health effects associated with prolonged exposure to high-intensity electromagnetic radiation in residential environments [1,2]. To mitigate the impact of electromagnetic waves, two primary approaches are employed: surface-absorbing coatings and the utilization of the intrinsic absorption properties of concrete. Compared with conventional absorbing coatings, imparting cement-based materials with inherent absorption capability—achieved by incorporating functional phases with absorbing properties—overcomes challenges such as performance degradation due to poor durability and weak interfacial bonding [3]. Enhancement of the electromagnetic wave absorption performance of cement-based materials is typically achieved through structural modifications, such as introducing pore structures or incorporating electrically functional materials [4]. Pore formation not only optimizes the effective dielectric constant of cement composites to improve impedance matching [5], but also increases electromagnetic wave attenuation via enhanced internal and diffuse reflection [6]. Functional additives are commonly used in cement-based absorbing materials, including conductive materials (e.g., carbon black, graphene, carbon fibers) [7], dielectric materials (e.g., ZnO, TiO_2_, MnO_2_) [8], and magnetic materials (e.g., Fe_3_O_4_, steel, nickel-based compounds) [9]. These additives dissipate electromagnetic energy through mechanisms such as polarization and magnetic loss [4]. It is noteworthy that while current design strategies have significantly improved the absorption performance of cement-based materials, the introduction of pore structures inevitably reduces mechanical strength, and the high cost of functional additives remains a major barrier to large-scale application.

The development of CO_2_ curing technology not only offers a promising approach for the sequestration of greenhouse gases but also provides a reliable method for enhancing the strength and durability of cement-based materials [9]. The reaction of CO_2_ with alkalis such as Ca(OH)_2_ or alkaline minerals (e.g., 3CaO·SiO_2_, 2CaO·SiO_2_) to form stable carbonates (Equations (1)–(3)) plays a pivotal role in improving both the early-age and long-term strength of these materials [10]. Microstructural investigations have revealed that CO_2_ curing promotes the transformation of cement hydration products from low-density calcium silicate hydrate (C–S–H) to a high-density phase, thereby improving the microporous structure. Additionally, the in situ formation of nano-calcium carbonate (calcite phase) effectively fills pores within the cement matrix, leading to a reduction in total porosity [11]. These microstructural modifications collectively enhance the mechanical performance of cement-based materials. For instance, CO_2_ treatment has been shown to increase the compressive strength of cement mortar by 28.1% [12], raise the elastic modulus of recycled aggregate concrete by 10.8–25.0% [13], and improve the compressive strength of lightweight concrete by 17% [14]. Collectively, these findings demonstrate that CO_2_ curing technology represents an effective strategy for significantly improving the mechanical properties of cement-based materials.

Ca(OH)_2_ + CO_2_ → CaCO_3_ (1)

3CaO·SiO_2_ + 3CO_2_ + nH_2_O → 3CaCO_3_ + 3SiO_2_·nH_2_O (2)

2CaO·SiO_2_ + 2CO_2_ + nH_2_O → 2CaCO_3_ + 2SiO_2_·nH_2_O (3)

Reducing the cost of functional additives remains a key challenge in the engineering application of cement-based absorbing materials. Among these additives, dielectric and magnetic materials—capable of converting electromagnetic wave energy into heat through dielectric loss and magnetic loss mechanisms—are the most critical [15]. However, industrial-grade dielectric materials are often expensive, which limits the large-scale application of cement-based microwave-absorbing materials. Luckly, industrial production generates substantial quantities of solid waste, much of which contains dielectric components. These wastes therefore provide abundant, low-cost raw materials for the development of cement-based microwave-absorbing materials. For instance, carbon black, comprising 15–35% of rubber formulations, is added to enhance wear resistance and extend service life [16,17], while metal smelting slag is rich in transition metal oxides (TMOs) [18,19]. It is worth noting that the disposal of such wastes, including waste rubber and smelting slag, always faces significant environmental challenges. Therefore, utilizing solid waste materials with dielectric properties as functional additives offers a dual benefit: it enhances the dielectric loss performance of cement-based microwave-absorbing materials while simultaneously reducing production costs. Moreover, this approach provides an environmentally sustainable pathway for managing industrial solid waste.

**Figure 1:**
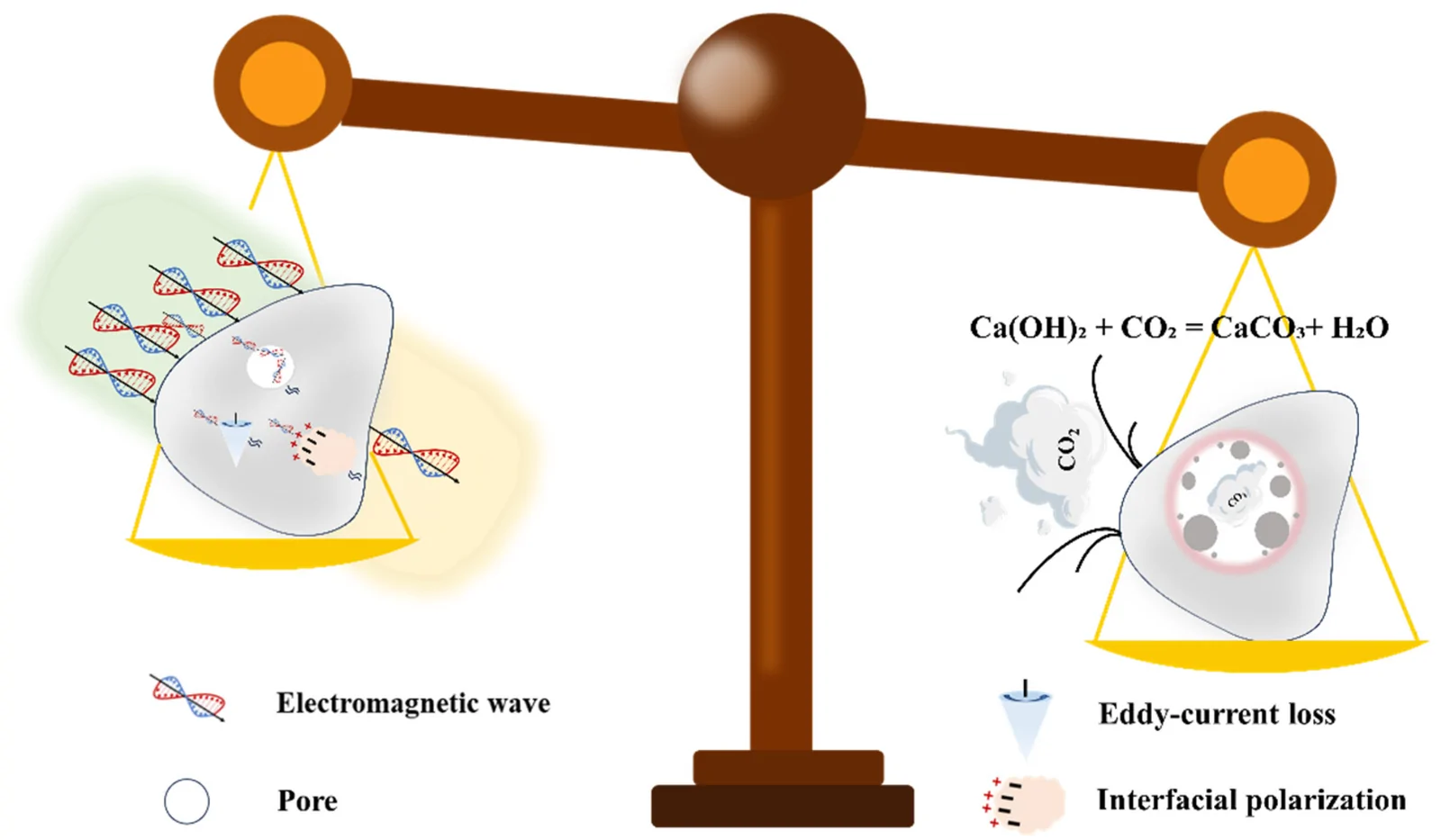
Structural design strategies for cement-based microwave-absorbing materials.

Building on the potential of CO_2_ curing technology to improve the mechanical performance of cement-based materials and the feasibility of incorporating solid waste to enhance microwave absorption, this study proposes a design strategy for a high-performance, low-cost cement-based microwave-absorbing material (Figure 1). The approach leverages carbon black derived from waste rubber powder and the abundant dielectric constituents in ironmaking slag to improve the material’s electromagnetic wave absorption capacity. When combined with CO_2_ curing, the microstructure and mechanical properties of the cement-based matrix are effectively tailored. This work not only delivers a high-performance cement-based microwave-absorbing material but also offers a sustainable pathway for the valorization of industrial solid waste.

## 2. Materials and Methods

### 2.1. Materials

Ordinary Portland cement (OPC) and standard sand were purchased from Shandong Shanshui Cement Co., Ltd. (Jinan, China) and Xiamen Ace Standard Sand Co., Ltd. (Xiamen, China), respectively. Rubber powder (1–3 mm in diameter) and slag (0.1–1.5 mm in diameter) were obtained from Dujiangyan Huayi Rubber Co., Ltd. (Dujiangyan, China) and Hebei Yanxi Mining Co., Ltd. (Shijianzhuang, China), respectively.

### 2.2. Preparation of Cement-Based Microwave-Absorbing Materials

The chemical composition of the cementitious microwave-absorbing materials was based on previous reports [20], with a mass ratio of cementitious material, standard sand, and water of 2:6:1. The chemical ratio of cement-based microwave-absorbing materials is shown in Table 1. Pure cement mortar, cement mortar with slag, cement mortar with rubber powder particles, and cement-based materials containing both rubber powder and slag were designated C, CS, CR, and CSR, respectively. After thorough mixing in a planetary cement-mortar mixer, the homogeneous mixture was cast into molds corresponding to the respective tests and kept under standard curing conditions (20 ± 1 °C, relative humidity ≥ 90%) for 24 h to ensure adequate initial hydration. The specimens were then demolded and transferred to two curing environments until the specified age: one group was stored in a constant-temperature and constant-humidity curing chamber (20 ± 1 °C, relative humidity of 70%) for standard curing, and the other in an accelerated carbonation chamber (CO_2_ concentration of 20%, 20 ± 1 °C, relative humidity of 70%) to simulate carbonation conditions.

### 2.3. Characterizations

X-ray diffraction (XRD; X’Pert PRO MPD, Malvern Panalytical, Almelo, The Netherlands) was employed to analyze the temporal evolution of the phases in the cement-based microwave-absorbing material, while scanning electron microscopy (SEM; Zeiss Gemini 300, Oberkochen, Germany) was used to examine its micromorphology.

### 2.4. The Fluidity Test

The fluidity was measured in accordance with the Method for Determination of Fluidity of Cement Mortar (JC/T 958-2005) [21,22]. A standard truncated-cone mold was used, with the following dimensions: height 60 ± 0.5 mm, upper inner diameter 70 ± 0.5 mm, and lower inner diameter 100 ± 0.5 mm. The mold was filled with fresh mortar, and the spread diameter was recorded within 6 min. Each group of samples was tested 5 times and the average value was taken.

### 2.5. Mechanical Properties Test

According to the “Test Method for Cement Mortar Strength” (GB/T 17671-2021) [23], compressive and flexural strength tests were conducted for mortar specimens at each sample [24]. After 28 days of curing, the specimens were tested using a compression testing machine (HUT-106D, Wance, Shenzhen, China) at a loading rate of 2.4 kN/s. Each group of samples was tested 5 times and the average value was taken.

### 2.6. Carbonization Depth Test

The carbonation depth was determined in accordance with the *Standard for Test Methods of Long-term Performance and Durability of Ordinary Concrete* (GB/T 50082-2009) [25] and *Testing of Concrete—Part 12: Determination of the Carbonation Resistance of Concrete—Accelerated Carbonation Method* (ISO 1920-12:2015) [26,27]. During specimen preparation, one surface was left exposed, while the remaining surfaces were sealed with heated paraffin wax. The specimens were then placed in a carbonation-curing chamber for accelerated testing. At carbonation ages of 1, 3, 7, and 28 days, specimens were fractured, and a 1% phenolphthalein–alcohol solution was sprayed onto the freshly exposed section. The carbonation depth was measured as the perpendicular distance from the color-change interface to the exposed surface.

### 2.7. Pore Structure Analysis of Cement-Based Materials

The pore structure of cement-based materials was characterized using the Nano Voxel 2000 X-ray 3D microscopy system (Tianjin Sanying Precision Instrument Co., Ltd., Tianjin, China.). This system offers a pixel resolution of 1 μm, a spatial resolution of 3 μm, and a planar detector imaging area of 244 mm × 195 mm, with a pixel matrix of 1920 × 1536. Following a 20 min preheating period, a cubic specimen measuring 20 mm × 20 mm × 20 mm was positioned for scanning. CT data were acquired using Nano Voxelscan software (SC500 V3.0.0) and subsequently reconstructed with Voxelstudio Recon (Version 1.0). Grid extraction was performed via Dragonfly software (Version 2022), and selected 3D image segments were converted into binary images. These binary datasets were then analyzed using the maximum sphere area algorithm implemented in Sypicore software (Version 2022) to obtain quantitative metrics of the pore structure.

### 2.8. Electromagnetic Parameters of Cement-Based Materials

Powdered hydrated cement, slag, and rubber were mixed with paraffin wax at a mass ratio of 2:1. The resulting mixture was placed in a mold and compressed at 10 MPa to form ring-shaped test specimens. The electromagnetic parameters of the cementitious materials were measured using a vector network analyzer (3656B, Electronics Technology Instruments Co., Ltd., Qingdao, China) over the frequency range of 2–18 GHz. The specimens had an inner diameter of 6.98 mm, an outer diameter of 3.04 mm, and a height of 2.00 mm. Three specimens from each group were tested, and the mean values were calculated.

### 2.9. Microwave Absorption Characterization of Cement-Based Materials

The microwave absorption performance of the cement-based materials was characterized using a vector network analyzer (VNA; model 3672B, China Electronics Technology Co., Ltd., Beijing China.) in an arch-reflection configuration. This method enabled direct measurement of the electromagnetic response of the specimens under the designated testing conditions. To meet the geometric requirements of the measurement system and ensure stable signal reflection, square specimens measuring 180 mm × 180 mm × 8 mm were prepared. For each material group, three specimens were tested under identical conditions, and the arithmetic mean of the measured values was used for subsequent analysis.

## 3. Results and Discussion

### 3.1. Physical and Chemical Structure of Cement-Based Microwave-Absorbing Materials

**Figure 2:**
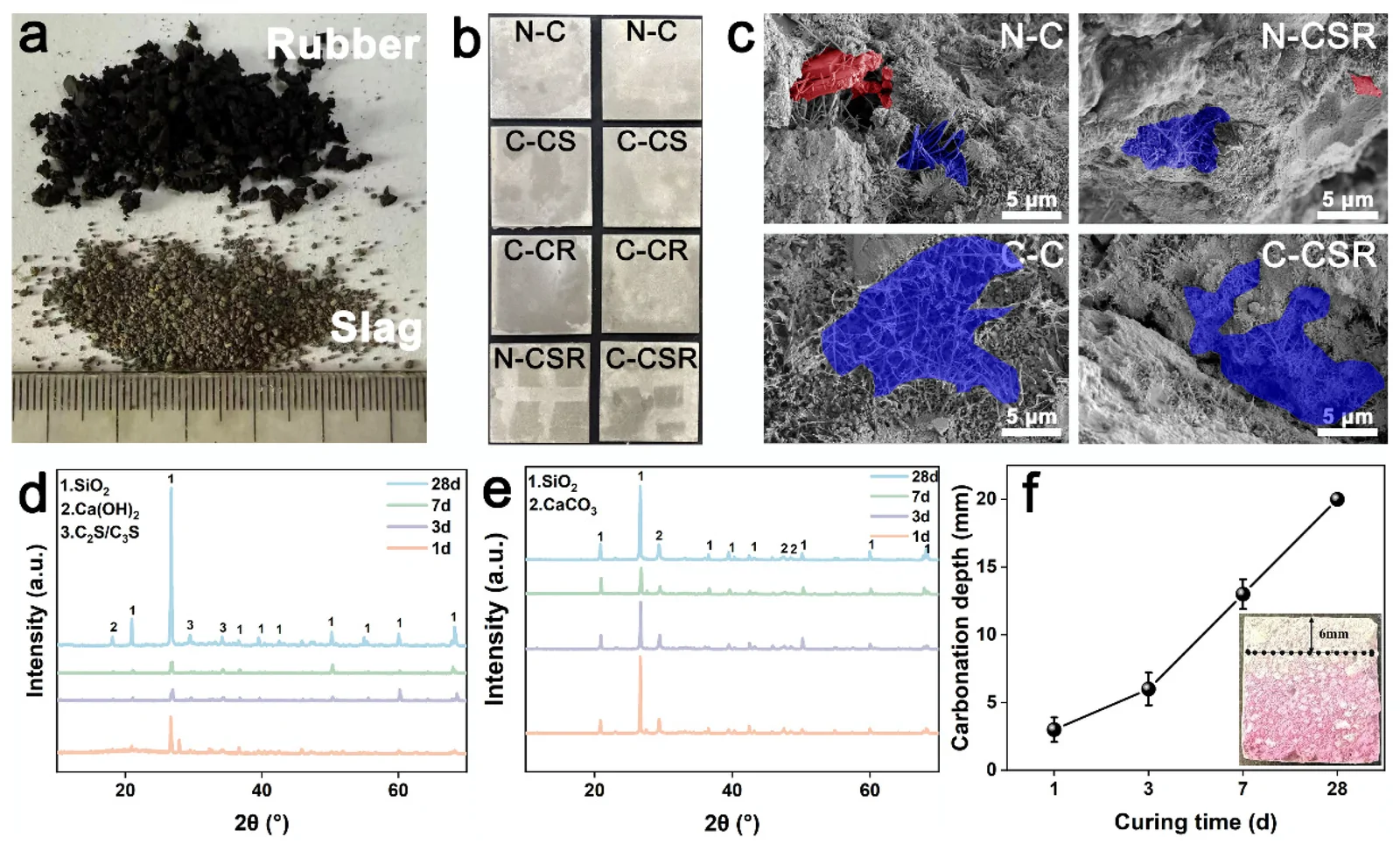
Physicochemical characteristics of cement-based materials. Optical images of (**a**) rubber powder and slag; (**b**) and (**c**) show optical images and the microstructure of cement-based microwave-absorbing materials, respectively; (**d**,**e**) show the XRD pattern of CSR cured in air and CO_2_; (**f**) variation in carbonation depth of cement over time under CO_2_ curing.

Figure 2a presents a digital photograph of the functional phases—rubber powder and slag—used in the cement-based microwave-absorbing material. X-ray fluorescence (XRF) analysis (Table S1) indicates that slag primarily consists of elements such as Si, Ti, Ca, Al, Fe, and Mn. The XRD pattern of the slag (Figure S1) shows characteristic diffraction peaks corresponding to CaTiO_3_, CaMgSi_2_O_6_, Fe_2_O_3_, CaCO_3_, and SiO_2_, indicating the presence of abundant transition-metal-containing phases. Notably, CaTiO_3_ and Fe_2_O_3_ are recognized as high-performance dielectric materials, and their presence is expected to enhance the electromagnetic wave absorption capability of the cement matrix [28,29]. The incorporation of slag and rubber powder particles into the cement-based material has minimal impact on hydration heat evolution (Figure S2) and the initial and final setting times (Figure S3). These findings underscore the potential of rubber powder and slag as functional additives for improving the electromagnetic absorption performance of cement-based materials without compromising their fundamental hydration behavior.

Figure 2b displays the cement-based microwave-absorbing material after being cured in air and CO_2_. Further insights into the hydration products were obtained through scanning electron microscopy (Figure 2c). In the microstructure of the specimen cured in air (N-C, and N-CSR), distinct plate-like calcium hydroxide [Ca(OH)_2_] (highlighted in red) and needle-like hydrated calcium silicate (highlighted in blue) are clearly visible [30]. When considered alongside the hydration heat evolution and setting time data (Figures S2 and S3), these observations indicate that the incorporation of rubber powder and slag does not significantly affect the cement hydration process. Notably, compared to specimens cured in air (without CO_2_), needle-like hydrated calcium silicate (marked in blue) and small granular structures were still observed in the microstructure of CO_2_-cured samples.

To further investigate the effects of CO_2_ curing processes, X-ray diffraction (XRD) analysis was conducted to examine phase transformations during the cement hydration process. In the specimen cured in air (Figure 2d), distinct diffraction peaks corresponding to Ca(OH)_2_ (PDF# 72-0156) were consistently observed at 1, 3, 7, and 28 days. In contrast, the specimens (Figure 2e) cured in CO_2_ exhibited the disappearance of the Ca(OH)_2_ peak, accompanied by the emergence of a pronounced CaCO_3_ diffraction peak (PDF# 05-0586), indicating successful carbonation. Additionally, Figure 2f and Figure S4 illustrate the relationship between CO_2_ curing duration and carbonization depth. After 28 days, the cement-based material appears to have undergone nearly complete carbonation. During this process, the relatively weak Ca(OH)_2_ is converted into the stronger and more stable CaCO_3_. The resulting CaCO_3_ formation further reduces the porosity of the cement-based material, which may contribute to the enhancement of its mechanical properties [31].

### 3.2. Electromagnetic Wave Absorption Performance Evaluation

**Figure 3:**
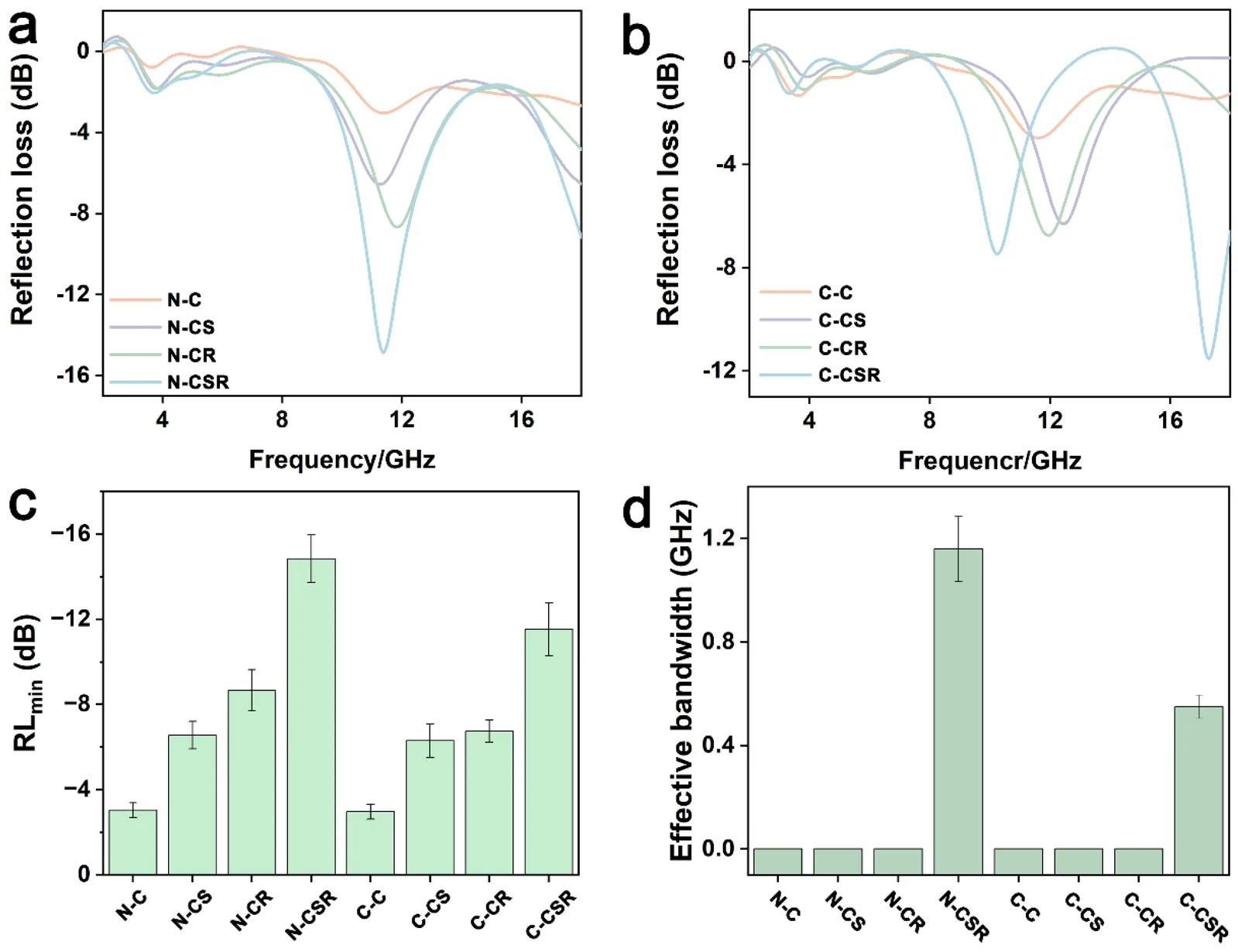
The calculated reflection loss for the cement-based microwave-absorbing materials cured in (**a**) air and (**b**) CO_2_. (**c**) Minimum reflection loss value and (**d**) effective absorption bandwidth of the specimen.

Figure 3 illustrates the microwave absorption performance of cement-based materials. The results reveal that incorporating rubber powder and slag increased the absorption capabilities of these materials, whereas CO_2_ curing exerts a somewhat detrimental effect on their performance. Reflection loss (RL) measurements across the 2–18 GHz frequency range indicate that the inclusion of rubber powder and slag consistently lowers RL values within the 8–12 GHz band, under both cured in air (Figure 3a) and CO_2_ conditions (Figure 3b).

Quantitative analysis (Figure 3c) further demonstrates that rubber powder contributes more substantially to RL reduction than slag. Specifically, RL values for N-CS and C-CS are −6.6 and −6.3, respectively, while those for N-CR and C-CR are −8.6 and −6.7, respectively. Notably, the combined use of rubber powder and slag yields a synergistic effect, with RL values reaching −14.85 dB and −11.53 dB for N-CSR and C-CSR, respectively. Beyond reflection loss, effective bandwidth analysis corroborates these findings: N-CSR and C-CSR achieve the highest bandwidths of 1.16 GHz and 0.55 GHz under conventional and CO_2_ curing, respectively. When compared with previously reported cement-based absorbers (Figure S5), both N-CSR and C-CSR demonstrate superior electromagnetic wave attenuation performance.

**Figure 4:**
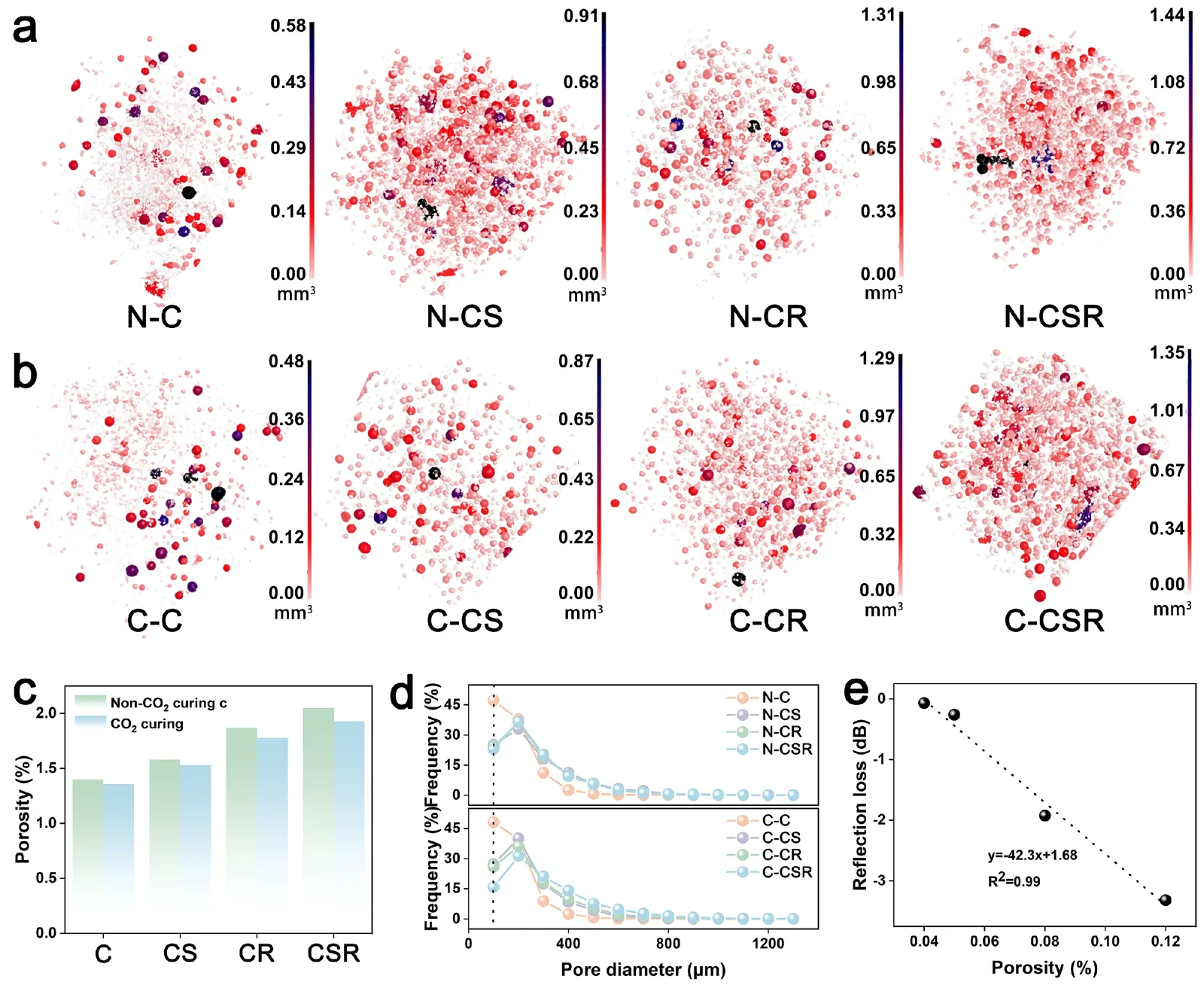
Three-dimensional reconstruction of the pore structures in cement-based materials cured in (**a**) air and (**b**) CO_2_ conditions. (**c**) Porosity and (**d**) pore size distribution of the cement specimens. (**e**) Correlation analysis between porosity variation and reflection loss difference.

In general, pore structure is a critical parameter influencing the reflection loss of cement-based microwave-absorbing materials [32]. Figure 4a,b present the three-dimensional reconstructions of pore structures in specimens cured in air and CO_2_, respectively, as obtained via CT scanning. The visualized models reveal that specimens incorporating slag and rubber powder exhibit a markedly higher pore count compared to pure cement specimens. This phenomenon is primarily attributed to the poor interfacial adhesion characteristics between the added phases (slag and rubber powder particles) and the cement slurry matrix [33].

Furthermore, the pore distribution density in specimens cured in CO_2_ is noticeably lower than that in air counterparts, resulting in a more compact microstructure. This observation aligns with previous findings indicating that calcium carbonate formed during CO_2_ curing tends to fill existing pores, thereby densifying the material [34]. Quantitative porosity analysis (Figure 4c) supports these conclusions: the porosities of N-C, N-CS, N-CR, and N-CSR were 1.40%, 1.58%, 1.87%, and 2.05%, respectively, while those of C-C, C-CS, C-CR, and C-CSR were slightly lower at 1.36%, 1.53%, 1.78%, and 1.93%, respectively.

In addition to overall porosity, pore size distribution analysis (Figure 4d) indicates that the incorporation of slag and rubber powder predominantly increases the proportion of pores exceeding 200 μm. These findings suggest that the combined use of slag and rubber powder effectively modifies and enhances the pore structure of cementitious materials.

It is noteworthy that the incorporation of slag and rubber powder simultaneously alters both the porosity and reflection loss characteristics of cement-based materials. To quantitatively assess the relationship between porosity and electromagnetic wave absorption performance, the differences in porosity and reflection loss under conventional (air) and CO_2_ curing conditions were analyzed through fitting (Figure 4e). The results reveal a linear correlation between porosity variation and the change in electromagnetic wave attenuation within the material. This behavior is primarily attributed to improved impedance matching facilitated by the porous structure, which enhances scattering and multiple scattering losses of electromagnetic waves in the cement matrix [5]. Because waste rubber particles, cement hydration products, and slag are incompatible with each other, the simultaneous addition of waste rubber powder and slag to cement creates more interfaces with weaker interactions, ultimately leading to an increase in the porosity of cement-based materials. These findings demonstrate that the addition of slag and rubber powder particles effectively modifies the pore structure of cement-based materials, leading to increased reflection loss and, consequently, improved electromagnetic wave absorption performance.

### 3.3. Electromagnetic Wave Dissipation Mechanism

**Figure 5:**
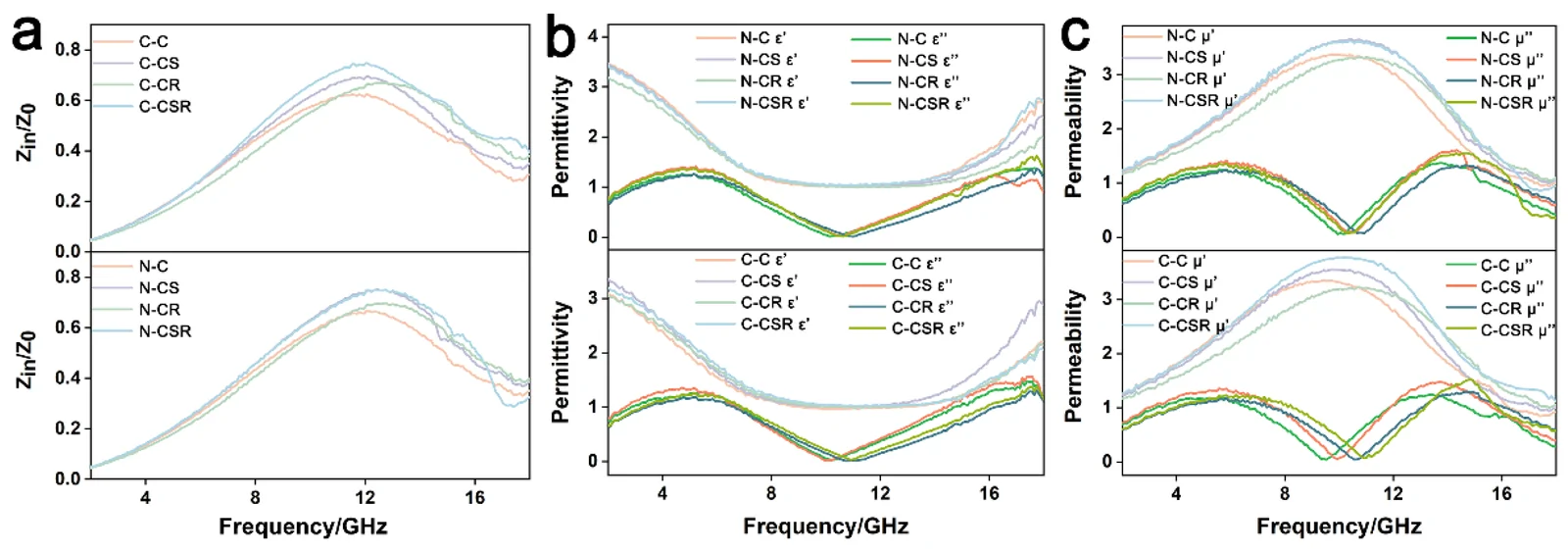
(**a**) Impedance matching, (**b**) complex permittivity, and (**c**) permeability of cement-based microwave-absorbing materials.

To elucidate the electromagnetic wave absorption mechanisms of cementitious materials, their impedance matching and electromagnetic parameters were systematically analyzed. Impedance matching, expressed as *Z_in_*/*Z*_0_, reflects the ability of incident electromagnetic waves to penetrate the material and serves as a critical metric for evaluating absorption performance [35]. As shown in Figure 5a, both specimens cured in air and CO_2_ exhibit high impedance matching values—approaching unity—within the 8–14 GHz frequency range, indicating strong absorption capabilities in this band. This observation aligns with the excellent absorption performance demonstrated in the 8–12 GHz range (Figure 3a,b). Importantly, specimens incorporating rubber particles (N-CR, C-CR) and slag (N-CS, C-CS) exhibit enhanced impedance matching, with *Z_in_*/*Z*_0_ values closer to 1. When considered alongside the improved absorption performance (Figure 3a,b), these results suggest that the inclusion of rubber particles and slag significantly contributes to the electromagnetic wave absorption efficiency of cementitious materials.

Beyond impedance matching, the dielectric constant for cement-based microwave-absorbing materials (Figure 5b) reveal that the real part (*ε*′) ranges from 1.1 to 3.5, while the imaginary part (*ε*″) spans 0.1 to 1.3. These values indicate that cement-based microwave-absorbing materials possess a measurable polarization capacity when subjected to electromagnetic fields [36]. Typically, *ε*′ characterizes the material’s ability to store electromagnetic energy, whereas *ε*″ reflects its capacity to dissipate that energy [37]. Analysis of the dielectric parameter curves shows that the incorporation of slag leads to an increase in *ε*″ in both N-CS and C-CS specimens, compared to N-C and C-C. A similar trend is observed in the magnetic permeability results, further reinforcing the conclusion. Collectively, these findings underscore the pivotal role of slag in enhancing the electromagnetic wave attenuation performance of cement-based materials.

**Figure 6:**
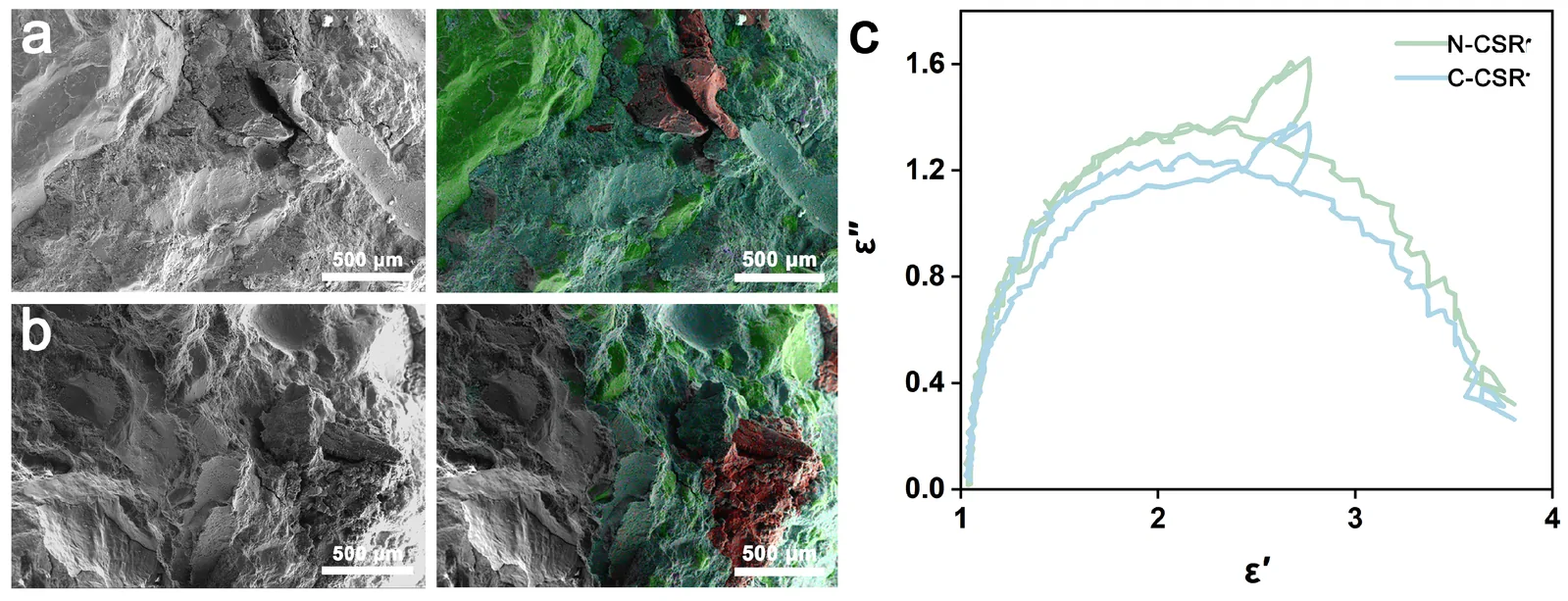
Micromorphology of (**a**) N-CSR and (**b**) C-CSR; (**c**) Cole–Cole plots of N-CSR and C-CSR.

Polarization loss represents one of the primary mechanisms underlying dielectric loss [38]. Cement-based materials such as N-CSR and C-CSR constitute a multiphase complex system (Figure 6a,b), comprising cement hydration products (hydrated calcium silicate and calcium hydroxide) as well as various composite phases (such as standard sand, rubber powder, and slag). Owing to disparities in lattice structures and dielectric properties among these constituents, pronounced heterogeneous interfaces are formed, which in turn give rise to significant interfacial polarization effects under electromagnetic wave irradiation. Cole–Cole plots (Figure 6c) of N-CSR and C-CSR reveal four distinct semicircular arcs, each corresponding to a Debye dipole relaxation process [39,40], thereby confirming the presence of substantial polarization loss within these cement-based absorbing materials. Notably, the incorporation of slag leads to an increased number of semicircles in the Cole–Cole plots of N-CS and C-CS compared to those of N-C and C-C (Figure S6). This observation suggests that slag addition effectively enhances the polarization loss characteristics of cementitious composites.

**Figure 7:**
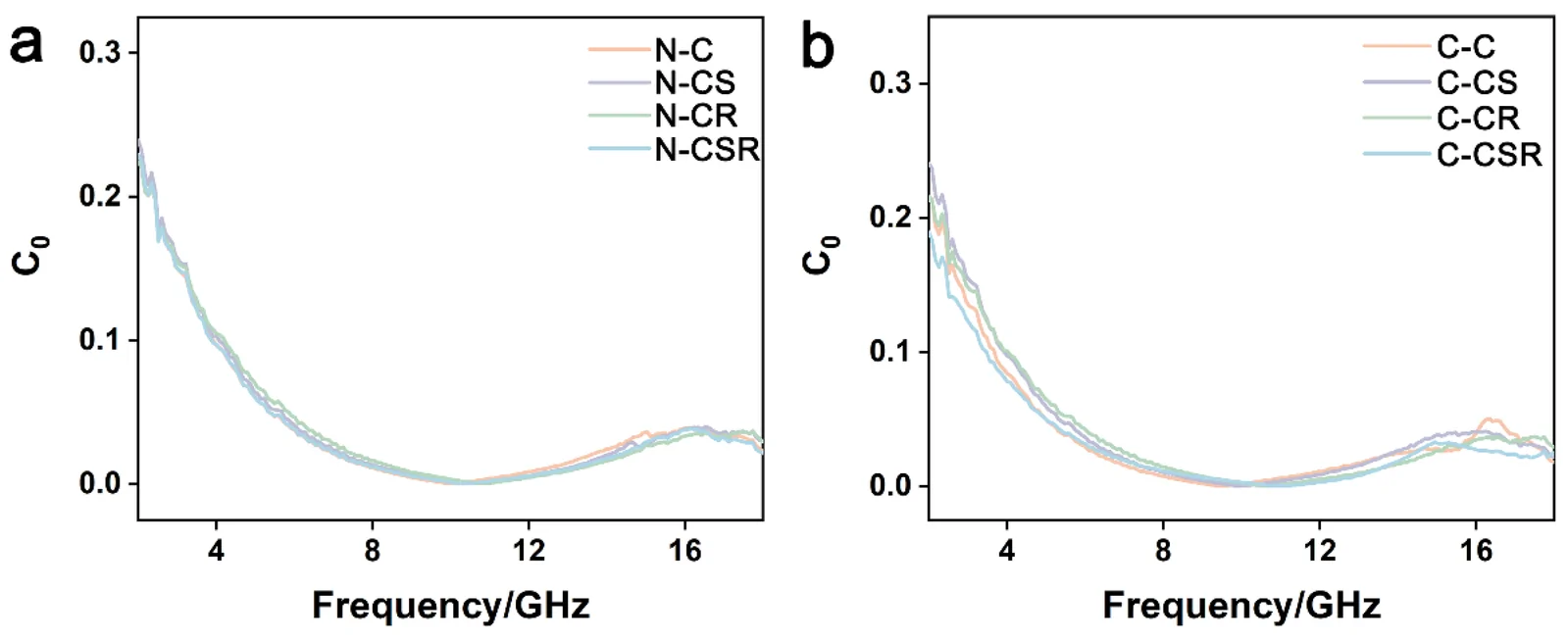
Eddy current loss (C_0_) of cement-based materials cured in (**a**) air and (**b**) CO_2_ conditions.

Magnetic loss in materials primarily arises from four mechanisms: eddy current loss, hysteresis loss, domain wall resonance, and ferromagnetic resonance. Among these, hysteresis loss is generally negligible, while domain wall resonance typically occurs in the low-frequency microwave region [41]. Consequently, eddy current loss and resonance loss are considered the dominant energy dissipation pathways in most practical applications [42]. The eddy current loss in cement-based materials can be quantitatively assessed using the parameter *C*_0_, defined as *C*_0_ (*C*_0_ = *μ*″(*μ*″) − 2*f* − 1 = 2*πμ*_0_*d*2*σ*), where *μ*_0_ is the vacuum permeability, *σ* is the electrical conductivity, and d is the sample thickness [43]. Notably, *C*_0_ remains relatively constant within the 8–12 GHz frequency range for both specimens cured in air (Figure 7a) and CO_2_ (Figure 7b), suggesting that eddy current loss is the predominant mechanism in this band [41]. Outside this range (particularly at lower and higher frequencies) resonance-related losses become more significant [44].

**Figure 8:**
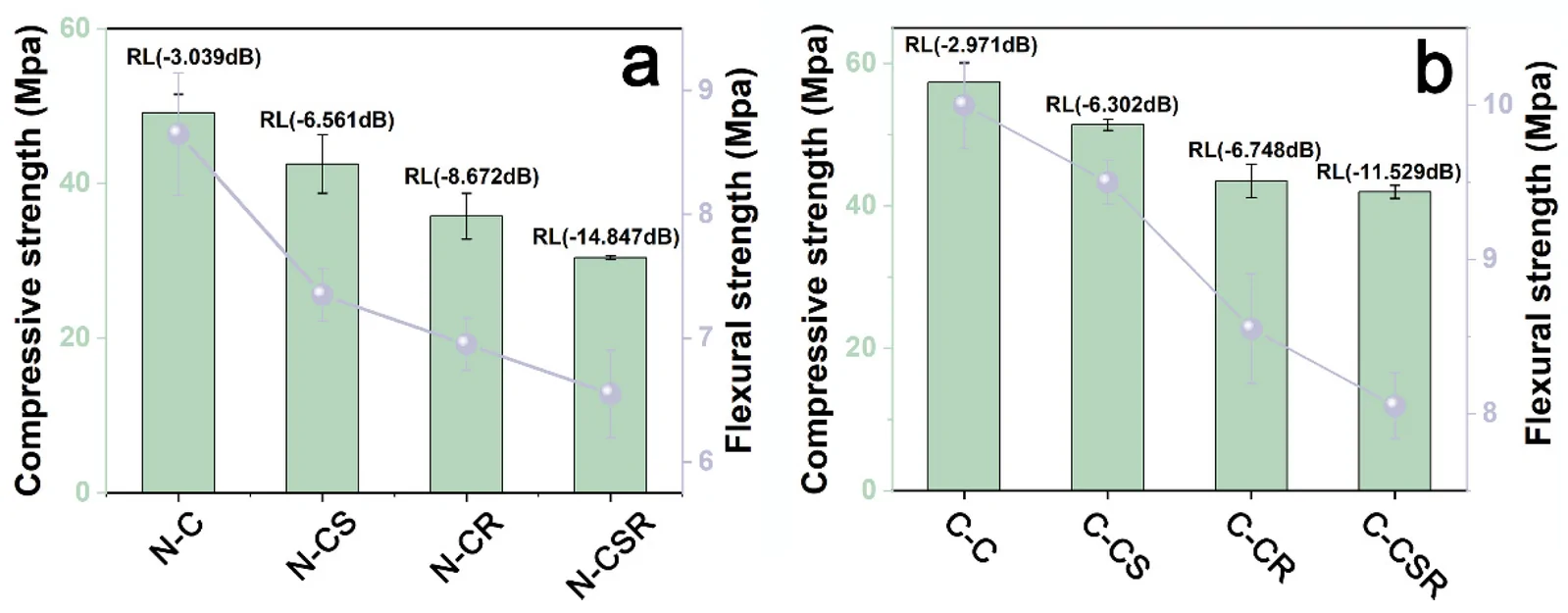
Mechanical properties of cement-based absorbing materials cured in (**a**) air and (**b**) CO_2_ conditions.

In addition to their electromagnetic wave absorption capabilities, the mechanical properties of cement-based absorbing materials are equally critical [45]. Notably, the incorporation of slag and rubber powder particles markedly diminished both the compressive strength (Figure 8a) and flexural strength (Figure 8b) of the composites, irrespective of whether conventional or CO_2_ curing methods were employed. This decline in mechanical performance is primarily attributed to the weak interfacial bonding between the introduced phases (slag and rubber powder) and the cement matrix [1]. While the inclusion of these additives compromises mechanical integrity, it simultaneously enhances the material’s electromagnetic wave absorption efficiency (Figure 8a,b), underscoring their functional significance.

Encouragingly, the CO_2_ curing process substantially mitigated the loss in mechanical strength. Specifically, the compressive and flexural strengths of the CO_2_-cured C-CSR increased by 38% and 23%, respectively, compared to the N-CSR cured in air. These findings demonstrate that CO_2_ curing offers an effective strategy to reconcile the trade-off between mechanical robustness and electromagnetic wave absorption, thereby providing a solid theoretical foundation for the development and practical deployment of high-performance absorbing concrete.

## 4. Conclusions

In short, this study presents the development of a high-performance cement-based microwave-absorbing material utilizing solid waste materials (rubber powder and slag) through targeted engineering of the material’s structural characteristics, including its chemical composition and pore architecture. The poor interfacial compatibility between rubber powder and cement paste was strategically leveraged to enhance the pore structure, particularly promoting the formation of macropores (>200 μm), thereby improving impedance matching. Additionally, the incorporation of slag significantly boosted the dielectric and magnetic loss capabilities of the composite. By simultaneously tuning the pore morphology and electromagnetic parameters, the cement-based absorbing materials demonstrated markedly improved microwave attenuation performance. Notably, the reflection loss values of the N-CSR and C-CSR samples increased by 2.8-fold and 1.8-fold, respectively, compared to their N-C and C-C counterparts. Furthermore, the integration of CO_2_ curing technology enhanced the mechanical strength of the material (achieving a 38% increase in compressive strength and a 23% increase in flexural strength) without compromising its electromagnetic absorption efficiency. Overall, this work not only introduces a robust design strategy for cement-based absorbers but also offers a sustainable pathway for the valorization of industrial solid waste.

## Figures and Tables

**Table 1:** Chemical ratio of cement-based microwave-absorbing materials.

Sample ID	Cement (g)	Standard Sand (g)	Rubber (g)	Slag (g)	Curing
**N-C**	100	300	0	0	Air
**N-CS**	100	270	0	30	Air
**N-CR**	100	300	8	0	Air
**N-CSR**	100	270	8	30	Air
**C-C**	100	300	0	0	CO_2_
**C-CS**	100	270	0	30	CO_2_
**C-CR**	100	300	8	0	CO_2_
**C-CSR**	100	270	8	30	CO_2_

## Data Availability

The original contributions presented in this study are included in the article/Supplementary Materials. Further inquiries can be directed to the corresponding author.

## Supplementary Materials

The following supporting information can be downloaded at https://www.mdpi.com/article/10.3390/polym18161942/s1: Table S1 chemical composition of the slag; Figure S1. The X-ray diffraction spectrum of the slag; Figure S2. Hydration heat analysis of cement-based microwave absorbing materials (a) heat flow, (b) cumulative heat release; Figure S3. Setting time of cement-based microwave absorbing materials; Figure S4. Carbonization depth of cement-based microwave absorbing materials; Figure S5. Comparison of reflection loss and effective absorption bandwidth of cement-based microwave absorbing materials; Figure S6. Cole-cole plots of (a) N-C; (b) N-CS; (c) N-CR; (d) C-C; (e) C-CS, and (f) C-CR. References [46,47,48,49,50,51] are cited in Supplementary Materials.
