# Supplementary material for: Carbonation-Cured Cementitious Materials Incorporating Waste Rubber/Slag with Balanced Mechanical Strength and Microwave Absorption Performance
Source: Polymers (Basel). 2026 Aug 7;18(16):1942. doi: 10.3390/polym18161942 (PMC13516380; doi:10.3390/polym18161942)
Supplement: Supplementary file 1 [file polymers-18-01942-s001.zip › polymers-4395038-supplementary.pdf]

Supporting information

Carbonation Cured Cementitious Materials Incorporating Waste Rubber/Slag with the Balanced Mechanical Strength and Microwave Absorption Performance

Xuemin Zeng [1,2], Hao Zhang [2], Hongping Zhang [1,3,*], Pan He [4], Laibao Liu [5], Xian Jian [6], Xiaoshuang Shi [7,*], Youhong Tang [8,*], Qingyuan Wang [3,7]

[1] State key Laboratory of Metal Mining Safety and Disaster Prevention and Control, Maanshan, Anhui, China

[2] Anhui University of Technology, Maanshan, Anhui, China

[3] School of Mechanical Engineering, Institute for Advanced Study, Chengdu University, Chengdu 610106, China

[4] Sichuan Provincial Engineering Research Center of Functional Development and Application of High Performance Special Textile Materials, Chengdu Textile College, Chengdu, 611731, China

[5] School of Materials and Chemistry, Southwest University of Science and Technology, Mianyang 621010, China

[6] School of Materials and Energy, University of Electronic Science and Technology of China, Chengdu 611731, China

[7] Failure Mechanics & Engineering Disaster Prevention and Mitigation, Key Laboratory of Sichuan Province, College of Architecture & Environment, Sichuan University, Chengdu 610065, China

[8] Institute for Nanoscale Science and Technology, College of Science and Engineering, Flinders University, South Australia 5042, Australia

* Correspondence: zhp1006@126.com (H Zhang), shixs@scu.edu.cn (X Shi) and youhong.tang@flinders.edu.au (Y Tang)

Table S1 chemical composition of the slag.

**Component**	SiO_2	TiO_2	CaO	Al_2O_3	MgO	Fe_2O_3	MnO_2	Others
**Content range (wt%)**	21.78	20.06	30.12	12.68	7.82	2.17	1.07	4.3

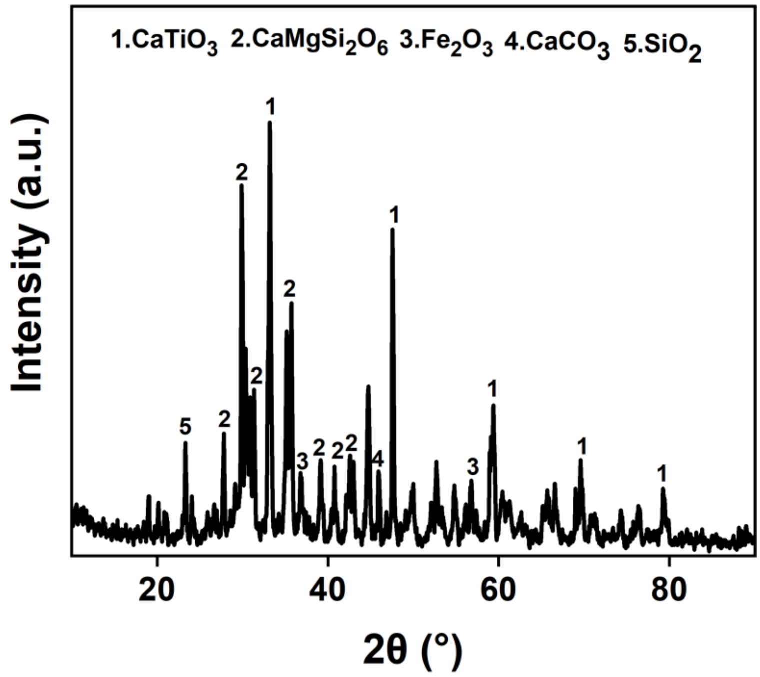

Figure. S1. The X-ray diffraction spectrum of the slag.

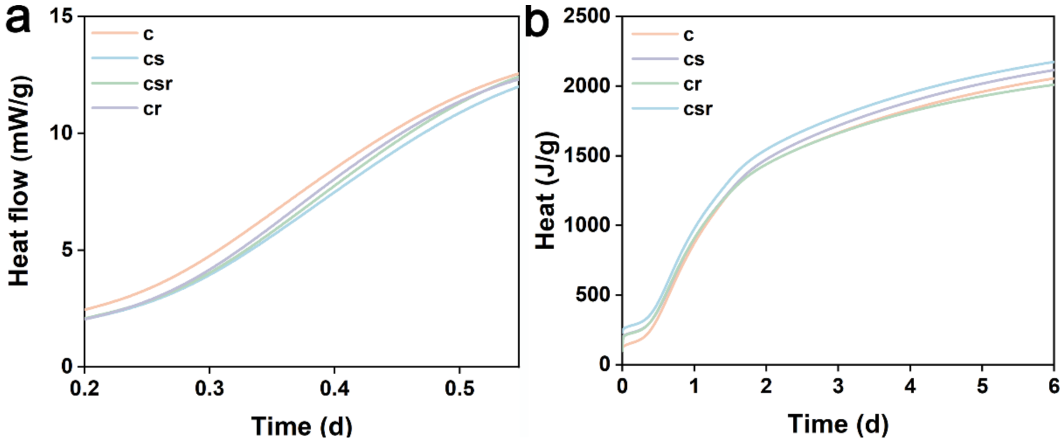

Figure. S2. Hydration heat analysis of cement-based microwave absorbing materials (a) heat flow, (b) cumulative heat release.

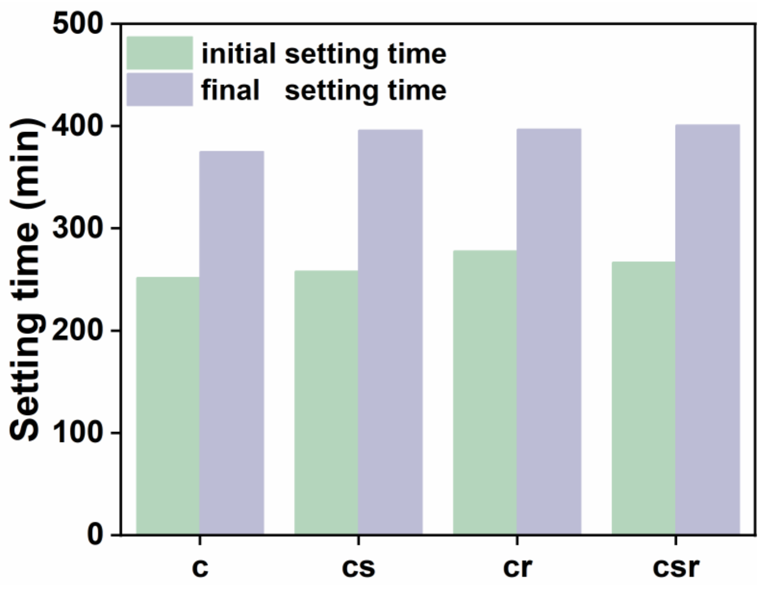

Figure. S3. Setting time of cement-based microwave absorbing materials.

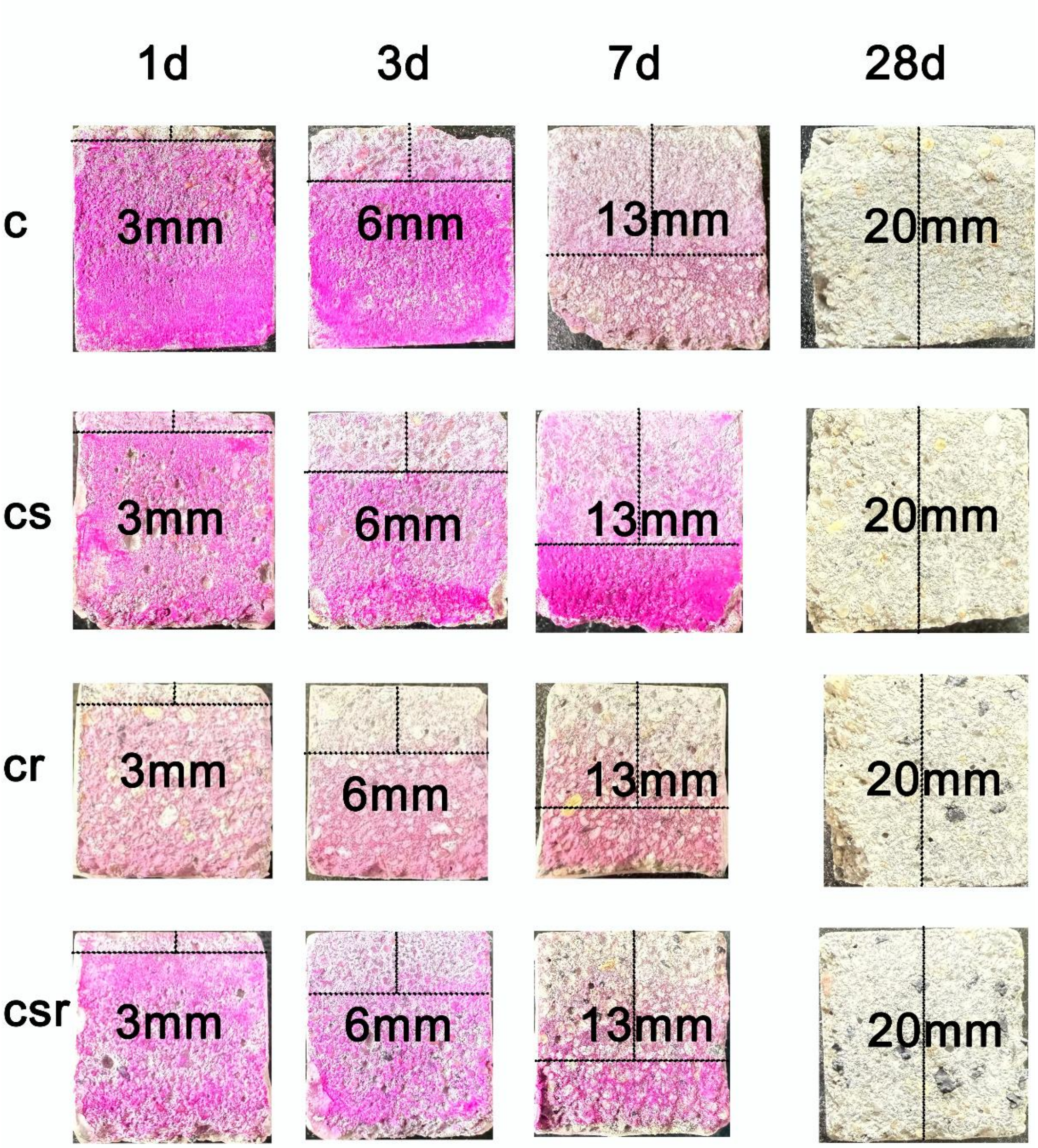

Figure. S4. Carbonization depth of cement-based microwave absorbing materials.

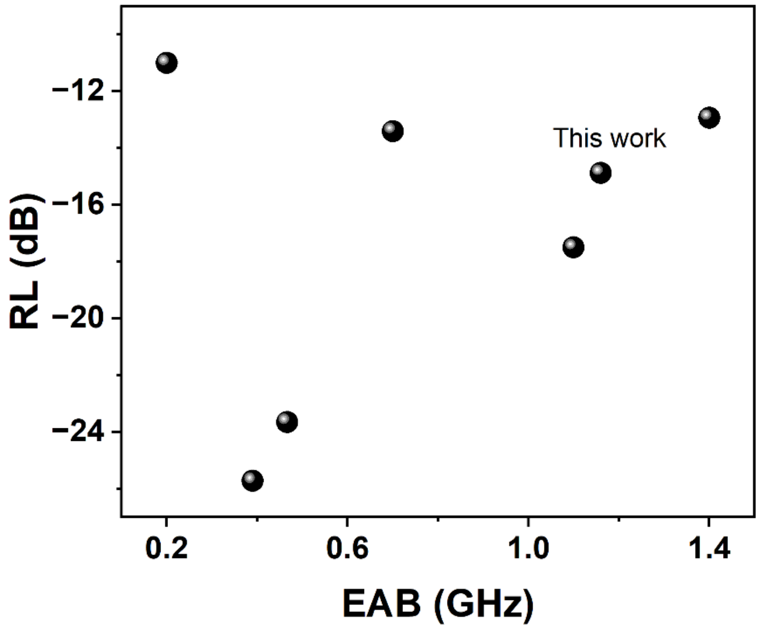

Figure. S5. Comparison of reflection loss and effective absorption bandwidth of cement-based microwave absorbing materials. [1-6]

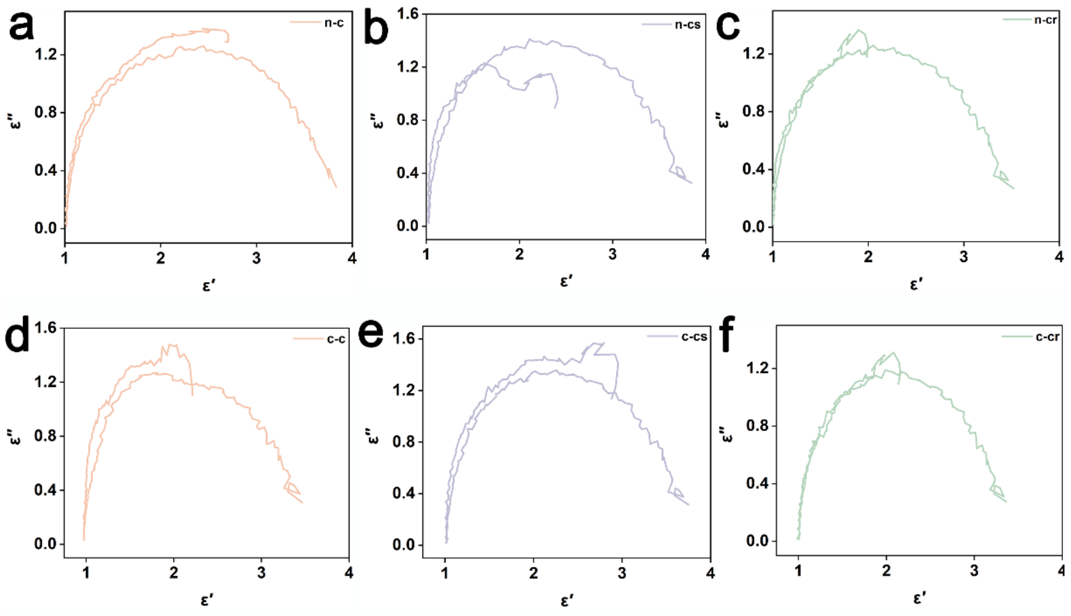

Figure. S6. Cole-cole plots of (a) N-C; (b) N-CS; (c) N-CR; (d) C-C; (e) C-CS, and (f) C-CR.

Reference

[1] Wang Z., Zhang T., Zhou L., Investigation on electromagnetic and microwave absorption properties of copper slag-filled cement mortar, *Cem. Concr. Compos.* **2016**, *74*, 174-181.

[2] Ren M., Li F., Gao P., Niu Y., Wei J., Yu Q., Design and preparation of double-layer structured cement-based composite with inspiring microwave absorbing property, *Constr. Build. Mater.* **2020**, *263*, 120670.

[3] Li H., Chen H., Wei M., Wang R., Lei N., Wang Q., Design and preparation of double-layer iron ore tailings cement-based electromagnetic wave absorbing materials containing carbon black and steel fiber, *Constr. Build. Mater.* **2023**, *364*, 129972.

[4] Liu X., Wang N., Wang Z., Effects of the absorber, thickness and surface roughness on the electromagnetic wave absorption characteristics of magnesium phosphate cement, *Constr. Build. Mater.* **2022**, *344*, 128149.

[5] Wang T., Ning X., Yang L., Rao F., Jiang K., Electromagnetic wave absorption properties of red mud-blast furnace slag based geopolymers filled with various wave-absorption materials, *Constr. Build. Mater.* **2023**, *404*, 133335.

[6] Cui X., Sun S., Han B., Yu X., Ouyang J., Zeng S., Ou J., Mechanical, thermal and electromagnetic properties of nanographite platelets modified cementitious composites, *Compos. Part. A-Appl. S.* **2017**, *93*, 49-58.
